# AHNAK suppresses tumour proliferation and invasion by targeting multiple pathways in triple-negative breast cancer

**DOI:** 10.1186/s13046-017-0522-4

**Published:** 2017-05-12

**Authors:** Bo Chen, Jin Wang, Danian Dai, Qingyu Zhou, Xiaofang Guo, Zhi Tian, Xiaojia Huang, Lu Yang, Hailin Tang, Xiaoming Xie

**Affiliations:** 1 Department of Breast Oncology, Sun Yat-Sen University Cancer Center, State Key Laboratory of Oncology in South China, Collaborative Innovation Center for Cancer Medicine, 651 East Dongfeng Road, Guangzhou, 510060 People’s Republic of China; 2 College of Pharmacy, University of South Florida, 12901 Bruce B Downs Blvd, MD30, Tampa, FL 33612-4749 USA

**Keywords:** AHNAK, Triple-negative breast cancer, AKT, MAPK, Wnt/β-catenin pathway

## Abstract

**Background:**

AHNAK, also known as desmoyokin, is a giant protein with the molecular size of approximately 700 kDa and exerts diverse functions in different types of cancer.

**Results:**

In the present study, we demonstrated that AHNAK mRNA levels were down-regulated in 7 out of 8 human breast cancer cell lines, especially in triple - negative breast cancer (TNBC) cell lines. Moreover, in patients with TNBC, the expression of AHNAK gene was inversely correlated with the tumor status (*P* = 0.015), lymph node status (*P* < 0.001), lymph node (LN) infiltration (*P* < 0.001) and TNM stage (*P* < 0.001). Moreover, down-regulated AHNAK expression was considered an independent prognostic factor associated with the poor survival of patients with TNBC. Overexpression of AHNAK in two TNBC cell lines, MDA-MB-231 and BT549, suppressed the in vitro TNBC cell proliferation and colony formation, and inhibited the in vivo TNBC xenograft growth and lung metastasis. The tumor suppressing effect of AHNAK in TNBC was associated with the AKT/MAPK signaling pathway and Wnt/β-catenin pathway. Consistent results were observed when AHNAK was knockdown in BT20 and MDA-MB-435 cells.

**Conclusions:**

Taken together, our results suggest that AHNAK acts as a tumor suppressor that negatively regulates TNBC cell proliferation, TNBC xenograft growth and metastasis via different signaling pathways.

**Electronic supplementary material:**

The online version of this article (doi:10.1186/s13046-017-0522-4) contains supplementary material, which is available to authorized users.

## Background

It is well known that breast cancer is a devastating disease with extensive intra-tumour and inter-tumour heterogeneity [1, 2]. Triple-negative breast cancer (TNBC), which lacks the expression of ER, PR and HER2 [3], is a unique subtype of breast cancer with limited treatment options and poor prognosis, accounting for 15–20% of breast cancers [4]. In China, although the overall incidence of breast cancer is lower than that in Western countries [5], the total number of patients with TNBC is relatively high. Despite advances in breast cancer treatment, the median overall survival (OS) for patients with TNBC is unfavourable compared with the mean OS for other breast cancer subtypes patients [6–8]. In this regard, it is necessary to investigate the molecular pathogenesis of TNBC and to explore novel therapeutic targets to improve the prognosis of TNBC patients.

AHNAK, also known as desmoyokin [9], is a large protein that was originally identified as a desmosomal plaque protein found at the periphery of the cytoplasmic plaque of desmosomes in the stratified squamous epithelia [10]. AHNAK has been previously reported to be expressed in several intracellular locations, including the plasma membrane, cytoplasm and nucleus [11]. Previous studies have indicated that AHNAK is involved in several important physiological activities, such as cardiac L-type Ca^2+^ channel function [12], neuronal cell differentiation and calcium signalling in T cells [13, 14]. In recent years, there has been increasing interest in understanding the function of AHNAK in various malignant tumours. So far, it has been demonstrated that the expression of AHNAK is variable in different types of cancer. For example, the expression of AHNAK is down-regulated in Burkitt lymphoma, small cell lung carcinoma and neuroblastoma [15, 16] but upregulated in glioma, mesothelioma, fibrosarcoma and prostate cancer [17]. Due to its large size and protein structure, AHNAK can facilitate the binding of multiple proteins and can mediate signalling events [18, 19]. The results of a recent study using a transgenic mouse model of breast cancer and human breast cancer samples suggest that AHNAK can act as a tumour suppressor that mediates the negative regulation of cell growth via the modulation of the TGFβ/Smad signalling pathway [20]. However, the expression profile of AHNAK in TNBC and its function have not been elucidated. In this study, we investigated the role of AHNAK in the pathogenesis of TNBC and assessed the effect of AHNAK on clinicopathological characteristics and prognosis by examining its expression in breast cancer cell lines and patient tissues and by characterizing its function in TNBC using both in vitro and in vivo models. We found that the expression of AHNAK is associated with the biological characteristics and prognosis of TNBC and the likelihood of lung metastasis. Moreover, the aggressive nature of TNBC with reduced expression of AHNAK was partly attributable to the activation of the AKT/MAPK and Wnt/β-catenin signalling pathways.

## Methods

### Clinical samples

This study was approved by the Ethics Committee of Sun Yat-Sen University Cancer Centre Health Authority. A total of 221 matched human triple-negative breast cancer (TNBC) tissues and 51 their adjacent normal mammary tissues (Normal 1), 20 non-triple-negative breast cancer (NTNBC) tissues and the corresponding paired normal adjacent tissues (Normal 2) were collected between October 2001 and September 2009 at Sun Yat-Sen University Cancer Centre. The details of the NTNBC samples are given in the supplementary information (Additional file 1: Table S1). The resected tissues were immediately cut and stored in RNAlater (Ambion). The collection and use of tissues followed procedures that are in accordance with the ethical standards formulated in the Declaration of Helsinki.

### Cell cultures and transfection

All the cell lines were obtained from the American Type Culture Collection (Manassas, VA, USA), including normal mammary epithelial cell lines (184A1, MCF-10A), human breast cancer cell lines (MDA-MB-231, BT549, BT-20, MCF-7, T47D, BT474, MDA-MB-435 and BT-483) and human embryonic kidney 293 T cells. All of the cell lines were passaged in our laboratory for less than six months and maintained according to the supplier’s instructions. The cell lines were found to be free of mycoplasma infection and their authenticity was verified by DNA fingerprinting before use.

Lentivirus-mediated AHNAK-expressing vector (EX-V0190-Lv122) and control plasmids were purchased from GeneCopoeia (Rockville, MD, USA). According to the manufacturer’s instructions, the AHNAK cDNA-containing plasmids were transfected into 293 T cells (1 × 10^6^) for 48 h to generate lentiviral particles. Lipofectamine® 2000 (Invitrogen Life Technologies, Carlsbad, CA, USA) was used. The control groups included the vector-transfected group (EX-NEG-Lv122). The viral supernatant was subsequently collected and used to infect MDA-MB-231 and BT549 cells. Seventy-two hours post-transfection, western blotting was performed to determine the transfection efficiency (Additional file 2: Figure S1A). BT-20 and MDA-MB-435 cells were used for transfection with AHNAK siRNA. Five microlitres of control or targeted siRNAs were transfected with Lipofectamine 2000 (Invitrogen) according to the protocol provided by Santa Cruz (sc-97060). The cells were grown for 48 h before assessing gene and protein knockdown efficiencies by western blotting (Additional file 2: Figure S1B).

### Quantitative RT-PCR analysis (qRT-PCR)

Total RNA was extracted from the cells using TRIzol reagent according to the manufacturer’s instructions (Invitrogen, Carlsbad, CA, USA). A NanoDrop ND-1000 instrument was used to determine the concentrations of the RNA samples. The integrity of RNA was assessed by electrophoresis on a denaturing agarose gel. Reverse transcription and qRT-PCR reactions were performed using a QuantiFast SYBR® Green RT-PCR Kit (QIAGEN, Germantown, MD, USA), which is a one-step RT-PCR kit. Each reaction was performed in triplicate. The primer sequences are given as follows: AHNAK: 5′-ATGCTCCAGGGCTCAACCT-3' (forward) and 5'-CGTGCCCCAACGTTAAGCTT-3' (reverse); β-actin: 5'-CGCGAGAAGATGACCCAGAT-3' (forward) and 5′-GGGCATACCCCTCGTAGATG-3' (reverse); Wnt1: 5'-ATGGGGCTCTGGGCGCTGTTG-3' (forward) and 5'-TCACAGACACTCGTGCAGTAC-3' (reverse); c-Myc: 5’-AGAAATGTCCTGAGCAATCACC-3' (forward) and 5’-AAGGTTGTGAGGTTGCATTTGA-3' (reverse); β-catenin: 5′- CCGCATGGAAGAAATAGTTGAAG-3′ (forward) and 5′- CAATTCGGTTGTGAACATCCC-3′ (reverse). The real-time PCR assays were performed with the Bio-Rad IQTM5 Multicolour Real-Time PCR Detection System (USA). The specificity of the amplification products was confirmed by melting curve analysis. The values were normalized to internal controls and fold changes were calculated through relative quantification (2^-ΔΔCt^).

### Cell proliferation assay

Cells were seeded on 6-well plates at the desired cell concentrations. The numbers of cells were counted after 1, 2, 3 and 4 days of incubation using a Coulter Counter (Beckman Coulter, Fullerton, USA) in triplicate.

### Colony growth assays

Six-well plates were covered with a layer of 0.6% agar in medium supplemented with 20% foetal bovine serum. Cells were prepared in 0.3% agar and seeded in triplicate. Then the plates were incubated in a CO_2_ incubator at 37 °C for two weeks. Crystal violet was used to stain the colonies, and the colonies were counted.

### Immunohistochemistry

Immunohistochemistry (IHC) staining was performed as described previously [21]. The concentration used for AHNAK (Santa Cruz Biotechnology) was 1:50.

### Western blot

Briefly, total protein was extracted and separated by 10% sodium dodecyl sulfate polyacrylamide gel electrophoresis (SDS-PAGE) and then transferred onto PVDF membranes. The protein bands were probed with antibodies against AHNAK (Santa Cruz Biotechnology), Akt, phospho-Akt (Ser473), ERK1/2, phospho-ERK1/2 (Tyr202/Y204), phospho-c-Raf (Ser296), phospho-MEK1/2 (Ser221) (Cell Signaling Technology, Beverly, MA), c-myc, Wnt-1 and β-actin (Abcam, Cambridge, UK) overnight at 4 °C followed by incubation with HRP-conjugated second antibodies (Santa Cruz, CA, USA) (1:3500) and detected by enhanced chemiluminescence. The dilutions used for the anti-AHNAK and anti-β-actin antibodies were 1:200 and 1:5000, respectively. The dilution used for the other antibodies was 1:1000. β-actin was used as the protein-loading control.

### Mouse xenograft model

All the animal procedures were performed in accordance with institutional guidelines. Ethical approval was obtained from the Institute Research Ethics Committee of Sun Yat-sen University Cancer Center. MDA-MB-231 or BT549 cells were stably transfected with AHNAK or vector and collected and suspended in PBS at a concentration of 1 × 10^7^ cells/ml. Then, 200 μl of cancer cell suspension was subcutaneously inoculated into the dorsal flanks of nude mice ((female, 4–6 w; five in each group) using 1-ml syringes. The tumour size was measured every four days. After 28 days, the mice were euthanized, and the tumours were weighed. The tumour volumes were determined according to the following formula: A × B^2^/2, where A is the largest diameter and B is the diameter perpendicular to A [22]. To assay the effect of AHNAK on tumour metastasis, 1 × 10^5^ MDA-MB-231 or BT549 cells infected with AHNAK or vector were injected into the tail veins of nude mice (eight in each group). The cells were suspended in PBS at a concentration of 2 × 10^6^ cells/ml, and 50 μl of cancer cell suspension was injected into each mouse using a microsyringe. Necropsies were performed after 28 days. The numbers of microscopic metastases in the lungs per H&E-stained section from individual mice were determined.

### Bioinformatics analysis

The expression levels of the AHNAK transcript in breast cancers and normal breast tissues were determined from the Oncomine database (www.oncomine.org). The threshold was set at a two-fold difference in expression between cancers and normal tissues with a *P*-value < 0.01. The Cancer Genome Atlas (TCGA) [23] and METABRIC [24] datasets were analysed and the figures were generated using the cBio Cancer Genomics Portal (http://cbioportal.org) [25, 26]. All TCGA data included in this manuscript are in compliance with the TCGA publication guidelines.

### Statistical analyses

Statistical analyses were performed using the SPSS 16.0 software. Comparisons between groups were conducted with t-tests and χ^2^ tests. The Kaplan-Meier method was used to plot overall survival curves and relapse-free survival curves, and the log-rank test was used for comparison. Survival was calculated from the time of pathological diagnosis. Univariate and multivariate analyses (Cox proportional hazards regression model) were performed to identify the independent factors relevant to patient survival. The differences were considered statistically significant at *P* < 0.05.

## Results

### Bioinformatics analysis of AHNAK expression in human breast cancer

**Fig. 1:**
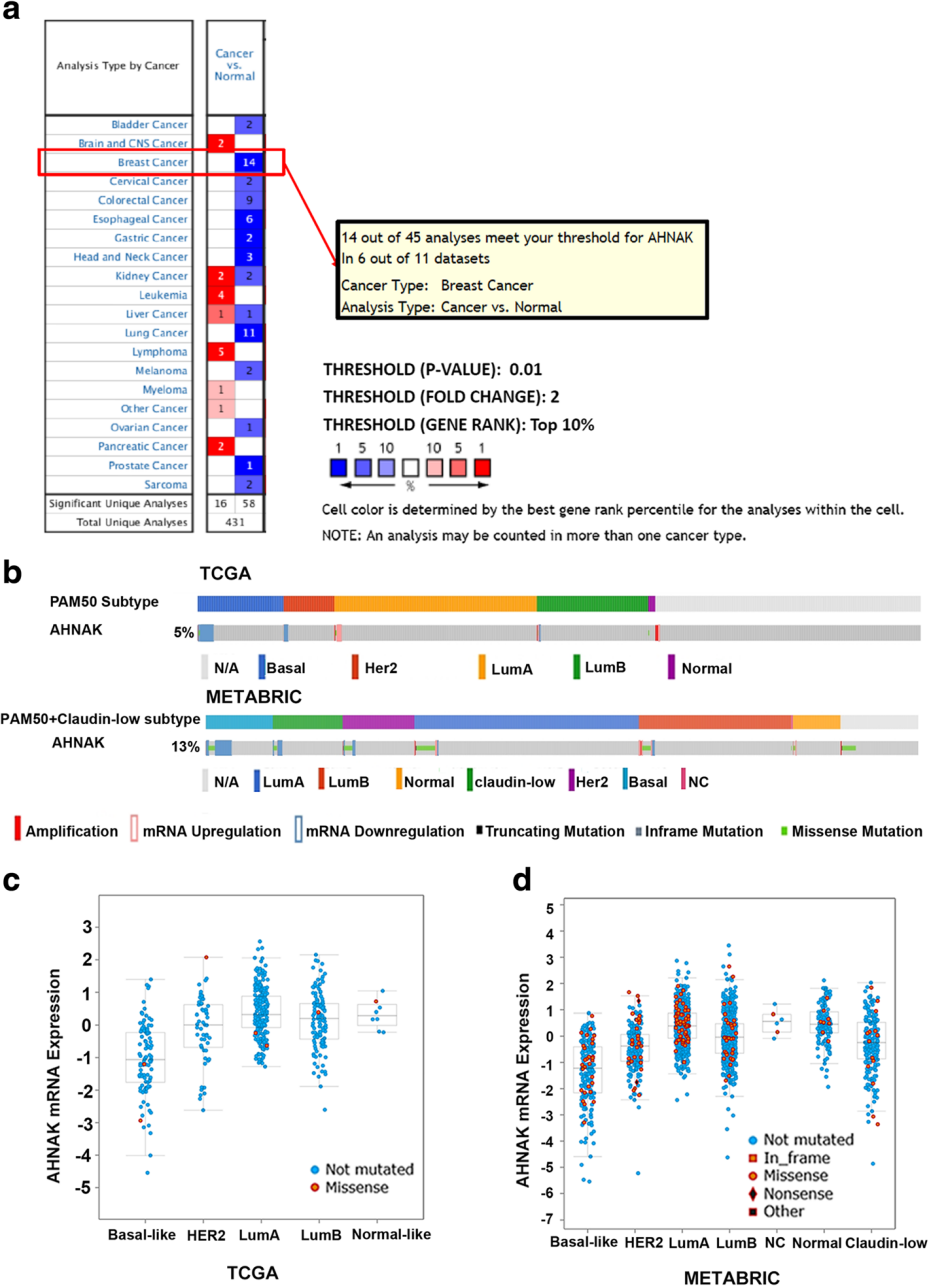
Bioinformatics analysis of AHNAK expression in human breast cancer. **a** Summary of AHNAK expression in cancers (Oncomine database). **b** The OncoPrint tab summarizes the genomic alterations of AHNAK across the sample set (TCGA and METABRIC). Each column represents a tumour sample. Plots showing AHNAK mRNA expression in tumours from TCGA (**c**) and METABRIC (**d**)

We searched the Oncomine database to get a general idea of the AHNAK expression levels in cancer tissues and normal tissues. The results showed that there were significant differences in the levels of AHNAK in different types of tumours (Fig. 1a). Among 20 common types of tumours, 12 tumour types exhibited decreases in AHNAK expression, including breast cancer. Six types of tumours showed increases and two types of tumours showed both decreases and increases in the expression of AHNAK. Then, we investigated the expression of AHNAK in TCGA and METABRIC, which have clinical data and information on gene expression and copy number variation (CNV) from approximately 3,000 patients. As Fig. 1b shows, most of the cases with down-regulated AHNAK belong to the basal-like classification (by PAM50 [27] and Claudin-low subtype [28]). Meanwhile, compared with the other subtypes of breast cancer, the basal-like subtype has the lowest average AHNAK expression (Fig. 1c and d).

### AHNAK expression was significantly down-regulated in triple-negative breast cancer and correlated with the clinicopathological characteristics and prognosis of TNBC patients

**Fig. 2:**
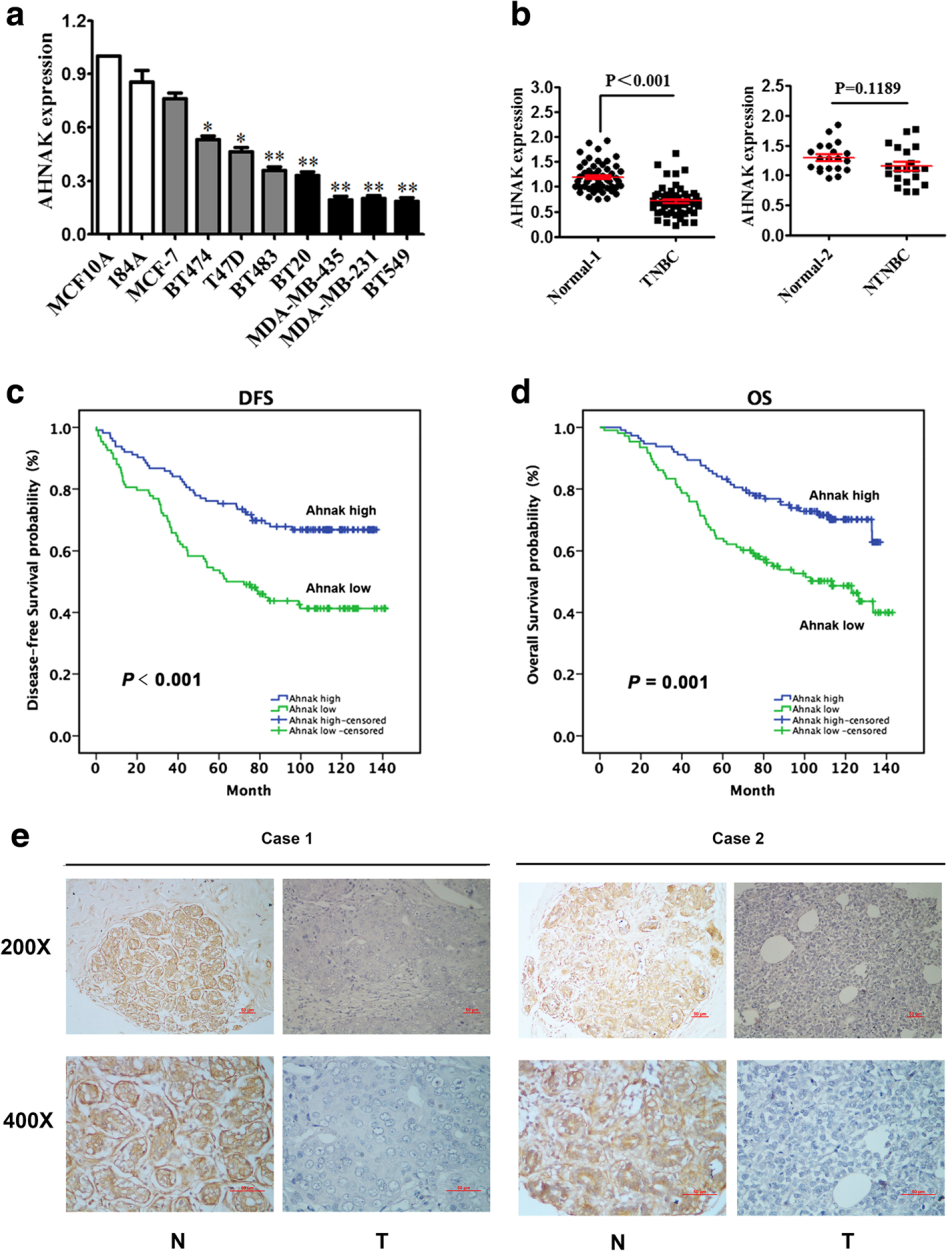
Expression of AHNAK is significantly down-regulated in triple-negative breast cancer. **a** AHNAK expression level determined by qRT-PCR in two normal mammary epithelial cell lines, four luminal breast cancer cell lines and four basal-like breast cancer cell lines. β-actin was used as a control for normalization. The error bars represent the standard deviation (SD). * *P* < 0.05 and **P < 0.01. **b** Expression levels of AHNAK in 51 TNBC specimens and the corresponding adjacent normal tissues (Normal 1) (*left*) and 20 NTNBC specimens and the corresponding adjacent normal tissues (Normal 2) (*right*). Low levels of AHNAK correlate with poor prognosis. **c** DFS curves for 221 TNBC patients with high or low AHNAK levels. **d** OS curves for 221 TNBC patients with high or low AHNAK levels (*right*). **e** Representative immunohistochemistry images of AHNAK expression in two TNBC cases

To further explore the expression of AHNAK in breast cancer, we tested the level of AHNAK mRNA in a panel of 10 breast cell lines, including 8 human breast cancer cell lines and 2 normal mammary epithelial cell lines using a qRT-PCR method. As Fig. 2a shows, compared with normal mammary epithelial cell lines, AHNAK mRNA levels are down-regulated in 7 human breast cancer cell lines, especially in TNBC cell lines (including BT20, MDA-MB-435, MDA-MB-231 and BT549). To confirm the results from the in vitro study, the levels of AHNAK mRNA were evaluated in 71 tissue samples, 51 of which were obtained from patients with TNBC and 20 from patients with non-TNBC (Fig. 2b). The results showed that the level of AHNAK mRNA was significantly reduced in TNBC samples, but no significant difference was found in the AHNAK mRNA levels between non-TNBC tissues and normal tissues. Moreover, in 221 cases of TNBC (including 51 cases of TNBC that we previously tested), we examined whether AHNAK expression was associated with clinicopathological parameters. Based on the mean AHNAK mRNA level, there were 108 patients with low AHNAK and 113 patients with high AHNAK expression. As shown in Table 1, the expression of AHNAK was inversely correlated with the tumour status (*P* = 0.015), lymph node status (*P* < 0.001), lymph node (LN) infiltration (*P* < 0.001) and TNM stage (*P* < 0.001) of TNBC patients. No significant correlation was found between AHNAK expression and other clinicopathological factors, including age, menopause, body mass index (BMI) and histological grade (*P* = 0.586, 0.338, 0.156 and 0.139, respectively). The results of the multivariate Cox regression analysis showed that patients with low levels of AHNAK had poor disease-free survival (DFS) and overall survival (OS) (Fig. 2c, *P* < 0.001 for DFS and Fig. 2d, *P* = 0.001 for OS; multivariate Cox regression analysis as shown in Table 2). Fig. 2e shows the representative immunohistochemical staining of AHNAK in matched TNBC tumour tissues and their corresponding non-tumour tissues.

**Table 1:** Association between AHNAK and clinicopathological characteristics in triple-negative breast cancer

Variables	Cases	Ahnak				*P* value
	(*n* = 221)	high	No. (%)	low	No. (%)	
Age (years)						0.586
<50	133	66	49.6%	67	50.4%	
≥50	88	47	53.4%	41	46.6%	
Menopause						0.338
yes	90	50	55.6%	40	44.4%	
no	131	63	48.1%	68	51.9%	
BMI						0.156
<25	169	91	53.8%	78	46.2%	
≥25	52	22	42.3%	30	57.7%	
Tumor status (T)						0.015*
T1	67	42	62.7%	25	37.3%	
T2	120	61	50.8%	59	49.2%	
T3	17	6	35.3%	11	64.7%	
T4	17	4	23.5%	13	76.5%	
Lymph node status (N)						<0.001*
N0	117	80	68.4%	37	31.6%	
N1	57	21	36.8%	36	63.2%	
N2	35	9	25.7%	26	74.3%	
N3	12	3	25.0%	9	75.0%	
Histological grade						0.139
G1 + G2	108	61	56.5%	47	43.5%	
G3	113	52	46.0%	61	54.0%	
LN infiltration						<0.001*
No	117	80	68.4%	37	31.6%	
Yes	104	33	31.7%	71	68.3%	
TNM stage						<0.001*
I	48	34	70.8%	14	29.2%	
II	111	64	57.7%	47	42.3%	
III	56	14	25.0%	42	75.0%	
IV	6	1	16.7%	5	83.3%	

*Abbreviation*: *BMI* body mass index, *LN* lymph node

*P < 0.05, statistically significant

**Table 2:** Prognostic value of AHNAK for overall survival in triple-negative breast cancer patients by Univariate and Multivariate analyses

Variables	Univariate analysis			Multivariate analysis		
	RR	95% IC	*P* value	RR	95% IC	*P* value
Age (<50 vs. ≥50 years)	1.137	0.748–1.728	0.548	-	-	-
Menopause (Yes vs. No)	1.069	0.701–1.630	0.758	-	-	-
BMI (<25 vs. ≥ 25)	1.057	0.654–1.710	0.820	-	-	-
Histological grade(G1 + G2 vs. G3)	1.626	1.064–2.485	0.025*	1.272	0.821–1.970	0.282
TNM Staging(I + II vs. III + IV)	3.340	2.199–5.073	<0.001*	2.834	1.827–4.397	<0.001
Ahnak(low vs. high)	2.094	1.366–3.209	0.001*	1.641	1.053–2.558	0.029

*Statistically significant prognostic factor identified by Univariate/Multivariate analysis

### AHNAK effected TNBC cell line proliferation and colony formation invasion

**Fig. 3:**
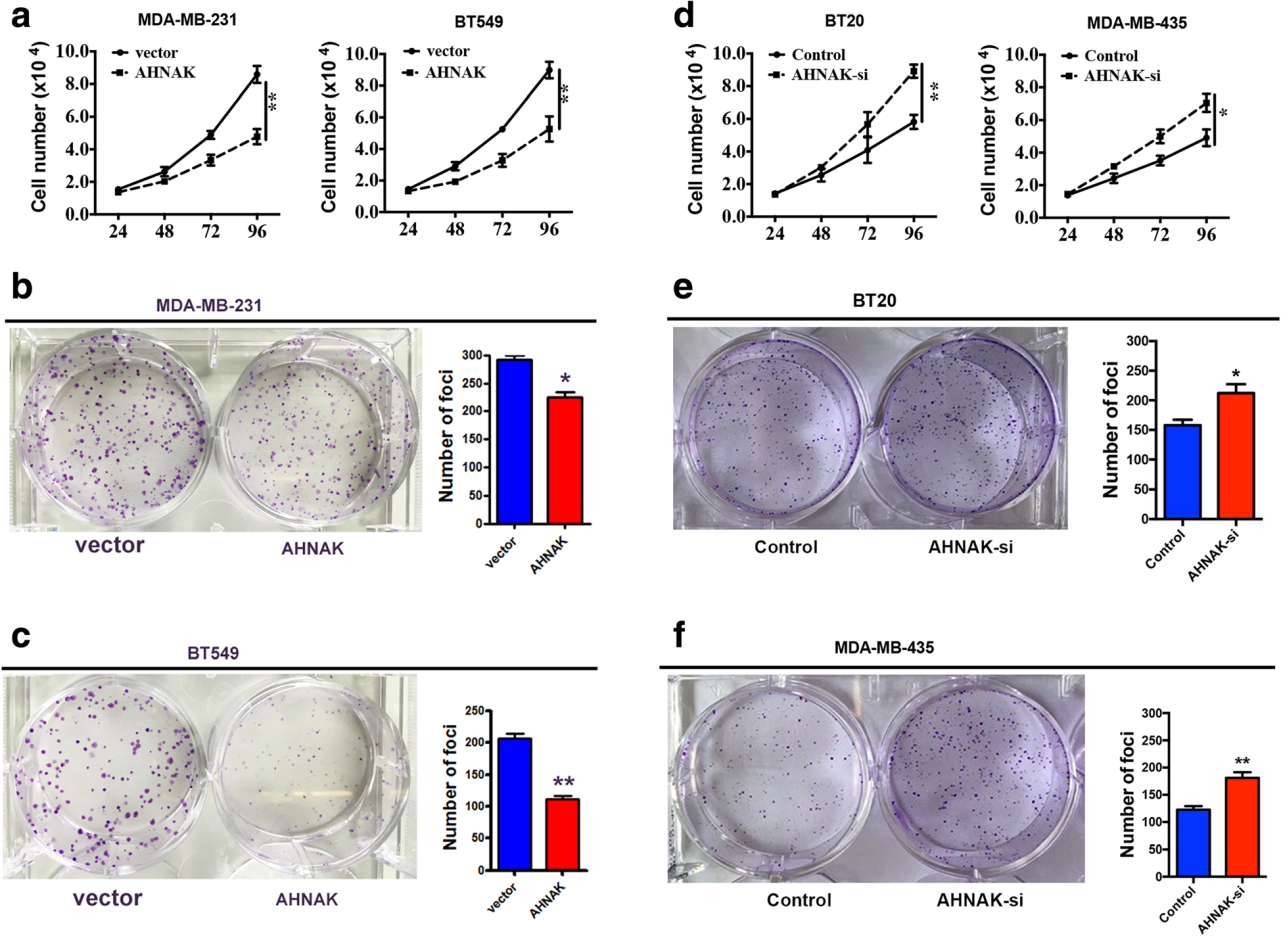
AHNAK inhibits proliferation and colony formation in TNBC cell lines. **a** The growth of MDA-MB-231 and BT549 cells infected with AHNAK-overexpressing or control vector was assayed by MTT. **P < 0.01. Colony formation assays performed on MDA-MB-231 (**b**) and BT549 (**c**) cells transfected with AHNAK or control vector. *P < 0.05 and **P < 0.01. **d** The growth of BT20 and MDA-MB-435 cells transfected with AHNAK siRNA or control was assayed by MTT. *P < 0.05 and **P < 0.01. Colony formation assays were performed on BT20 (**e**) and MDA-MB-435 (**f**) cells transfected with AHNAK siRNA or control. *P < 0.05 and **P < 0.01

Next, we studied the biological effects of AHNAK in TNBC. AHNAK-expressing vector was transfected into two TNBC cell lines, MDA-MB-231 and BT549. Compared with the control group, we found that the ectopic expression of AHNAK in MDA-MB-231 and BT549 cells could markedly inhibit cell proliferation (Fig. 3a) and colony formation (Fig. 3b and c). Furthermore, we used siRNA to perform knockdown of AHNAK expression in BT20 and MDA-MB-435 cells to assess the functional consequences. We found that knockdown of AHNAK expression could promote proliferation (Fig. 3d) and colony formation (Fig. 3e and f) of TNBC cells. The results thus suggest the role of AHNAK as a tumour suppressor in TNBC.

### Overexpression of AHNAK in TNBC cell lines inhibited in vivo tumour growth and lung metastasis

**Fig. 4:**
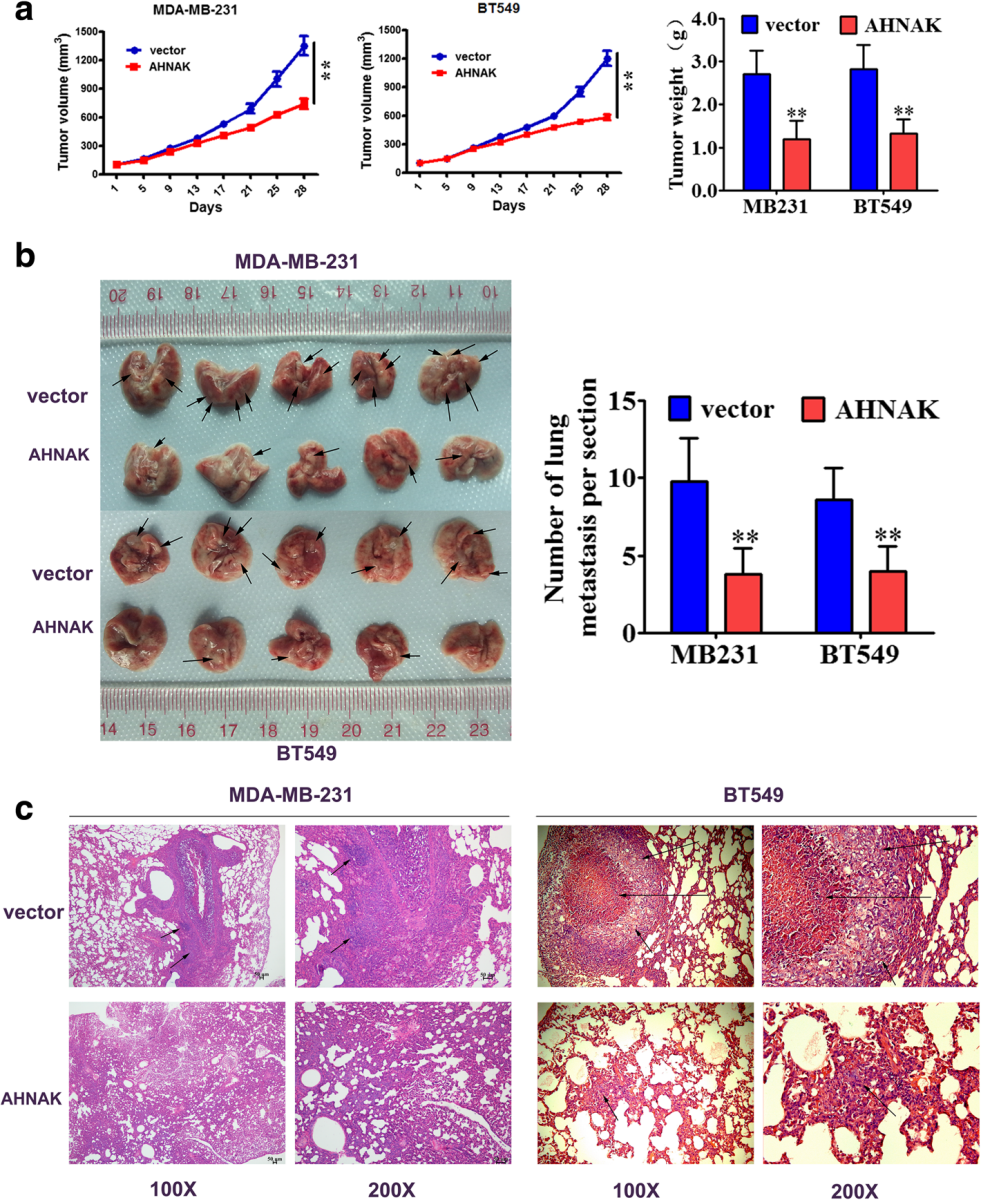
AHNAK inhibits TNBC growth and lung metastasis in vivo. MDA-MB-231 or BT549 cells infected with AHNAK or vector lentivirus were injected into the flanks of nude mice. **a** The growth curves of the tumours are plotted (left: MDA-MB-231; middle: BT549). The weights of the xenograft tumours are summarized in the right panel. All results are expressed as the mean ± SD of three independent experiments, *P < 0.05 and **P < 0.01. **b** Tumour metastasis in the mouse xenograft model. Metastatic nodules (arrows) on the lung surface. The number of nodules was quantified in the lungs of nude mice (*n* = 5 per group) 28 days after tail vein injection of AHNAK- or empty vector-transfected MDA-MB-231 and BT549 cells (**, *P* < 0.01, independent Student’s t-test). **c** The haematoxylin and eosin stained sections derived from metastatic nodules on the lung surface. Original magnification 100X and 200X

Based on the findings from the in vitro study and clinicopathological analysis, we adopted a xenograft model using human TNBC cells in nude mice to further verify the function of AHNAK in TNBC. As shown in Fig. 4a, compared with the control group, the mean size and weight of tumours of the AHNAK-overexpressing group were significantly lower. Next, we designed a cancer metastasis xenograft model by tail vein injection to assay the effect of AHNAK on tumour metastasis. Four weeks after injection, the mice were euthanized and their lungs were dissected. The metastatic nodules on the surface of the mouse lungs (arrows) were markedly decreased after overexpression of AHNAK (Fig. 4b). To confirm that the nodules were metastatic tumours, haematoxylin and eosin staining was used (Fig. 4c). The results indicated that AHNAK repressed TNBC proliferation and metastasis in vivo.

### AHNAK targets AKT/MAPK signalling and the Wnt/β-catenin pathway

**Fig. 5:**
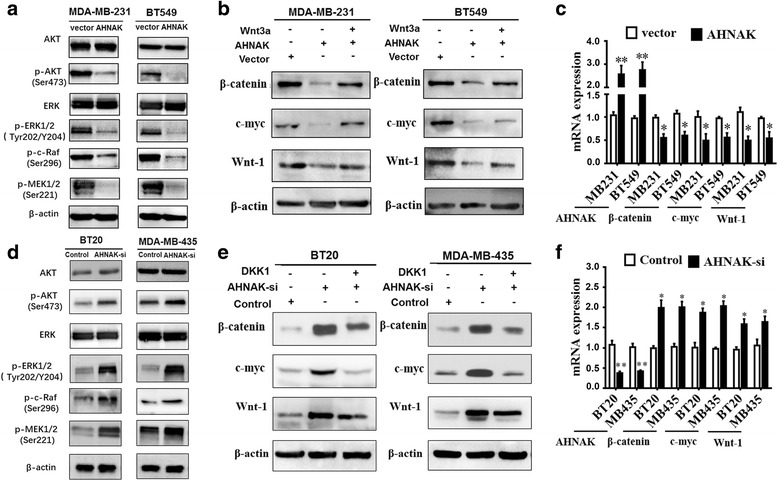
AHNAK targets the AKT/MAPK signalling pathway and the Wnt/β-catenin pathway. **a** Relative expression levels of AKT/MAPK signalling proteins were compared between the empty vector- and the AHNAK-expressing MDA-MB-231 and BT549 cells by western blotting. β-actin was used as the loading control. **b** Image of the blot shows that Wnt activator Wnt3a could reactivate the expression of β-catenin, c-myc, and Wnt1 expression in the MDA-MB-231 and BT549 cells infected with AHNAK-expressing vector. β-actin was used as the loading control. **c** In contrast to the corresponding vector-transfected groups, the levels of β-catenin, c-myc and Wnt-1 mRNA were significantly decreased in the MDA-MB-231 and the BT549 cells that were transfected with AHNAK-expressing vector.** d** Relative levels of AKT/MAPK signalling-related proteins were compared between the control and AHNAK siRNA-transfected BT20 and MDA-MB-435 cells by western blotting. **e** Image of the blot shows that Wnt inhibitor Dkk1 could effectively decrease the expression of β-catenin, c-myc, and wnt1 in the BT20 and MDA-MB-435 cells that were infected with AHNAK siRNA. **f** In contrast to the control group, the levels of β-catenin, c-myc and Wnt-1 mRNA were significantly increased in the BT20 and MDA-MB-435 cells that were transfected with AHNAK siRNA

As we found in vitro and in vivo, AHNAK partly inhibited TNBC cell growth and lung metastasis. Next, we wanted to identify the possible molecular mechanisms by which AHNAK regulates the biological characteristics of TNBC cells. We analysed the expression of a series of proteins likely to be affected by AHNAK. The results showed that the overexpression of AHNAK did not affect the total expression of AKT and ERK proteins but markedly suppressed the phosphorylation of AKT, ERK, Raf and MEK1/2 proteins in MDA-MB-231 and BT549 cells (Fig. 5a). These results suggested that AHNAK possibly promoted the growth of TNBC cells via the AKT/MAPK signalling pathway. In addition, we found that AHNAK expression partly regulated the Wnt/β-catenin pathway. According to results from previous studies, the Wnt signalling pathway is one of the key signalling pathways in cancer [29–31]. It is generally known that changes in cell motility and tumour metastasis are commonly related to the Wnt/β-catenin pathway. We used western blotting to detect the expression levels of Wnt/β-catenin pathway markers in AHNAK-modified cells. When AHNAK was overexpressed in MDA-MB-231 and BT549 cells, the western blot results confirmed that AHNAK could down-regulate β-catenin, c-myc and Wnt-1 (Fig. 5b). By contrast, the expression of these proteins in the AHNAK-overexpressing MDA-MB-231 and BT549 cells was decreased compared with the control, and Wnt3a could reactivate their expression (Fig. 5b). Moreover, the results from quantitative real time-PCR showed that, when AHNAK was overexpressed, the levels of β-catenin, c-myc and Wnt-1 mRNA were significantly decreased in MDA-MB-231 and BT549 cells (Fig. 5c). Overall, the results indicated that decreases in β-catenin, c-myc and Wnt-1 protein expression levels were most likely due to the reduced transcription of the corresponding mRNA. Meanwhile, consistent results were observed when AHNAK was knocked down in BT20 and MDA-MB-435 cells (Fig. 5d, e and f).

## Discussion

TNBC is a subtype of breast cancer that has some of the worst patient prognoses of all breast cancer subtypes and is not sensitive to normal endocrine therapy or targeted therapy against breast cancer [32]. Studies of molecular mechanisms in TNBC are extremely necessary. By mining the literature, we found that AHNAK is a very large protein that is involved in many cellular processes and pathways [33]. Down-regulation of AHNAK prevents cortical actin cytoskeleton reorganization. AHNAK forms a multimeric complex with actin and the Annexin 2/S100A10 complex at the plasma membrane, which suggests that AHNAK interacts with the cortical actin cytoskeleton as part of a submembranous complex [21]. Previous research also showed that AHNAK could potentiate TGFβ-induced transcriptional activity of R-Smad, which leads to the negative regulation of cell growth by stimulating the localization of Smad3 into the nucleus [20]. Meanwhile, cell- or tissue-specific processes or pathways regulated by AHNAK are quite distinct and are related to the cell or tissue type [33]. Although several proteins have been identified to interact with AHNAK, the function of AHNAK in breast cancer remains undefined.

Here, we demonstrated the functional significance of AHNAK in TNBC. Using public datasets from Oncomine (www.oncomine.org), the AHNAK mRNA level was found to be reduced in breast cancer, although there was a huge variation among different types of cancers. Meanwhile, both the TCGA and METABRIC datasets showed that the level of AHNAK mRNA was significantly decreased in the samples classified as basal-like (most TNBCs have basal-like characteristics). Consistent with previous findings [20], we found that the expression of AHNAK is low in several TNBC cell lines and that AHNAK might play a tumour suppressive role. In addition, we also found that AHNAK expression was markedly decreased in TNBC patient samples, and the expression of AHNAK is negatively correlated with some vital clinicopathological characteristics, such as tumour status (*P* = 0.015), lymph node status (*P* < 0.001), lymph node (LN) infiltration (*P* < 0.001) and TNM stage (*P* < 0.001) of TNBC, as well as prognosis of TNBC patients. From in vitro studies, we found that overexpression of AHNAK could inhibit proliferation and colony formation of TNBC cell lines. Conversely, knocking down AHNAK expression could promote the proliferation and colony formation of TNBC cell lines. As indicated, transfection with AHNAK-overexpressing vectors could decrease the growth and metastasis breast cancer xenografts. Thus, we confirmed the function of AHNAK in suppressing tumour progression.

In previous studies, AHNAK has been suggested to be involved in signalling pathways, such as the reorganization of the actin cytoskeleton network, the PI3K–PKB pathway to engage effector proteins, the formation of pseudopodial protrusions, and adaptation events to reprogram tumour cell biology [17, 34]. The Wnt/β-catenin signalling pathway plays critical roles in development and tissue homeostasis [35, 36]. As many previous studies have indicated, the Wnt/β-catenin signalling pathway also plays a vital role in breast cancer [37–39]. The role of Wnt signalling in primary TNBC and as a predictor of lung and brain metastasis has been described [40, 41]. A meta-analysis indicated that the Wnt pathway is activated in TNBC and that increased Wnt/β-catenin signalling is associated with metastatic disease and poor prognosis [41]. Notable Wnt transcriptional targets upregulated in TNBC include MYC [42], matrix metallopeptidase 7 (MMP7) [43], VEGF [44], MET [45], CD44 [46], snail (SNAI1) [47] and survivin (BIRC5) [48]. In this study, we explored the association between AHNAK and the Wnt/β-catenin signalling pathway and demonstrated that, in TNBC, AHNAK indeed regulated the expression of several important genes belonging to the Wnt/β-catenin signalling pathway, such as Wnt-1, β-catenin and c-myc, both at the mRNA level and at the protein level. Previous studies have confirmed that there is a link between AHNAK and c-myc. Overexpression of AHNAK could down-regulate c-myc and cyclin D1/D2, resulting in cell cycle arrest and growth retardation [20]. A recent study also showed that, in the absence of the ectopic expression of c-myc, the down-regulation of AHNAK could generate safer induced pluripotent stem cells (iPSCs) [49]. Moreover, we identified PI3K/AKT and MAPK/ERK as key signalling pathways involved in the inhibition of tumour cell proliferation mediated by AHNAK. The constitutive activation of PI3K/AKT and MAPK/ERK signalling pathways is an important event in breast cancer, as they regulate multiple cellular processes to promote cancer growth, survival, and metastasis [50, 51]. However, there are several limitations in our study that should be addressed. First, although we found that AHNAK affected some pathways to some extent, the specific details of these mechanisms are still unknown. In addition, it remains unclear whether the proposed role for AHNAK is limited to only the triple-negative subtype of breast cancer. Therefore, further studies will be needed to determine the exact function of AHNAK.

## Conclusions

In conclusion, our study demonstrates the potential tumour suppressive role and lung metastasis-inhibiting effect of AHNAK in TNBC. Moreover, AHNAK could, at least partially, affect the AKT/MAPK and the Wnt/β-catenin signalling pathways, which are important tumour-related signalling pathways in TNBC. Taken together, although more in-depth mechanisms and prognostic roles for AHNAK in TNBC need to be confirmed in the future, our findings provide a preliminary basis to explore AHNAK as a potential therapeutic candidate in TNBC.

## Additional files

Additional file 1: Table S1. Clinical information for 20 non-triple-negative breast cancer patients. (DOCX 16 kb)

Additional file 2: Figure S1. Assessment of transfection efficiency by western blotting. (TIF 398 kb)
