# Supplementary material for: AHNAK suppresses tumour proliferation and invasion by targeting multiple pathways in triple-negative breast cancer
Source: J Exp Clin Cancer Res. 2017 May 12;36:65. doi: 10.1186/s13046-017-0522-4 (PMC5427595; doi:10.1186/s13046-017-0522-4)
Supplement: Supplementary file 1 — Clinical information for 20 non-triple-negative breast cancer patients. (DOCX 16 kb) [file 13046_2017_522_MOESM1_ESM.docx]

**Supplementary Table1. Clinical information for 20 non- triple negative breast cancer patients.**

| **No.** | **Gender** | **Age(years)** | **Pathologic diagnosis** | **Tumor size（cm）** | **Positive lymph nodes** | **ER（%+）** | **PR（%+）** | **HER-2（+）** | **Ki67（%+）** | **Subtype** |
| --- | --- | --- | --- | --- | --- | --- | --- | --- | --- | --- |
| 1 | female | 38 | invasive ductal carcinoma | 1.8 | 0 | 20 | 1 | 2 | 60 | Luminal B |
| 2 | female | 68 | invasive ductal carcinoma | 6 | 0 | 0 | 0 | 3 | 30 | Her-2 |
| 3 | female | 58 | invasive ductal carcinoma | 2.3 | 0 | 10 | 0 | 3 | 7 | Luminal B |
| 4 | female | 59 | invasive ductal carcinoma | 3.5 | 3 | 60 | 90 | 0 | 10 | Luminal A |
| 5 | female | 57 | invasive ductal carcinoma | 4.5 | 0 | 0 | 0 | 3 | 30 | Her-2 |
| 6 | female | 48 | invasive ductal carcinoma | 1 | 0 | 95 | 95 | 0 | 10 | Luminal A |
| 7 | female | 39 | invasive ductal carcinoma | 4 | 1 | 50 | 90 | 1 | 20 | Luminal B |
| 8 | female | 51 | invasive ductal carcinoma | 2 | 0 | 70 | 80 | 1 | 40 | Luminal B |
| 9 | female | 54 | invasive ductal carcinoma | 2.6 | 1 | 0 | 0 | 3 | 5 | Her-2 |
| 10 | female | 49 | invasive ductal carcinoma | 5 | 8 | 80 | 2 | 2 | 20 | Luminal B |
| 11 | female | 30 | invasive ductal carcinoma | 4 | 1 | 30 | 1 | 1 | 40 | Luminal B |
| 12 | female | 55 | invasive ductal carcinoma | 2.5 | 9 | 20 | 20 | 2 | 30 | Luminal B |
| 13 | female | 47 | invasive ductal carcinoma | 3 | 0 | 90 | 90 | 3 | 20 | Luminal B |
| 14 | female | 45 | invasive ductal carcinoma | 3 | 0 | 80 | 95 | 0 | 10 | Luminal A |
| 15 | female | 41 | invasive ductal carcinoma | 3.2 | 4 | 20 | 0 | 3 | 10 | Luminal B |
| 16 | female | 69 | invasive ductal carcinoma | 4 | 0 | 90 | 90 | 0 | 10 | Luminal A |
| 17 | female | 48 | invasive ductal carcinoma | 4 | 10 | 60 | 90 | 2 | 30 | Luminal B |
| 18 | female | 64 | invasive ductal carcinoma | 2.8 | 0 | 80 | 80 | 1 | 20 | Luminal B |
| 19 | female | 42 | invasive ductal carcinoma | 2 | 0 | 85 | 90 | 3 | 15 | Luminal B |
| 20 | female | 47 | invasive ductal carcinoma | 3 | 2 | 70 | 90 | 1 | 60 | Luminal B |

ER, PR, Her-2 and ki67 were determined by immunohistochemistry.

Pathological was classified according to the American Joint Committee on Cancer (AJCC) 7th Edition.
