# Supplementary figures and images for: AHNAK suppresses tumour proliferation and invasion by targeting multiple pathways in triple-negative breast cancer
Source: J Exp Clin Cancer Res. 2017 May 12;36:65. doi: 10.1186/s13046-017-0522-4 (PMC5427595; doi:10.1186/s13046-017-0522-4)

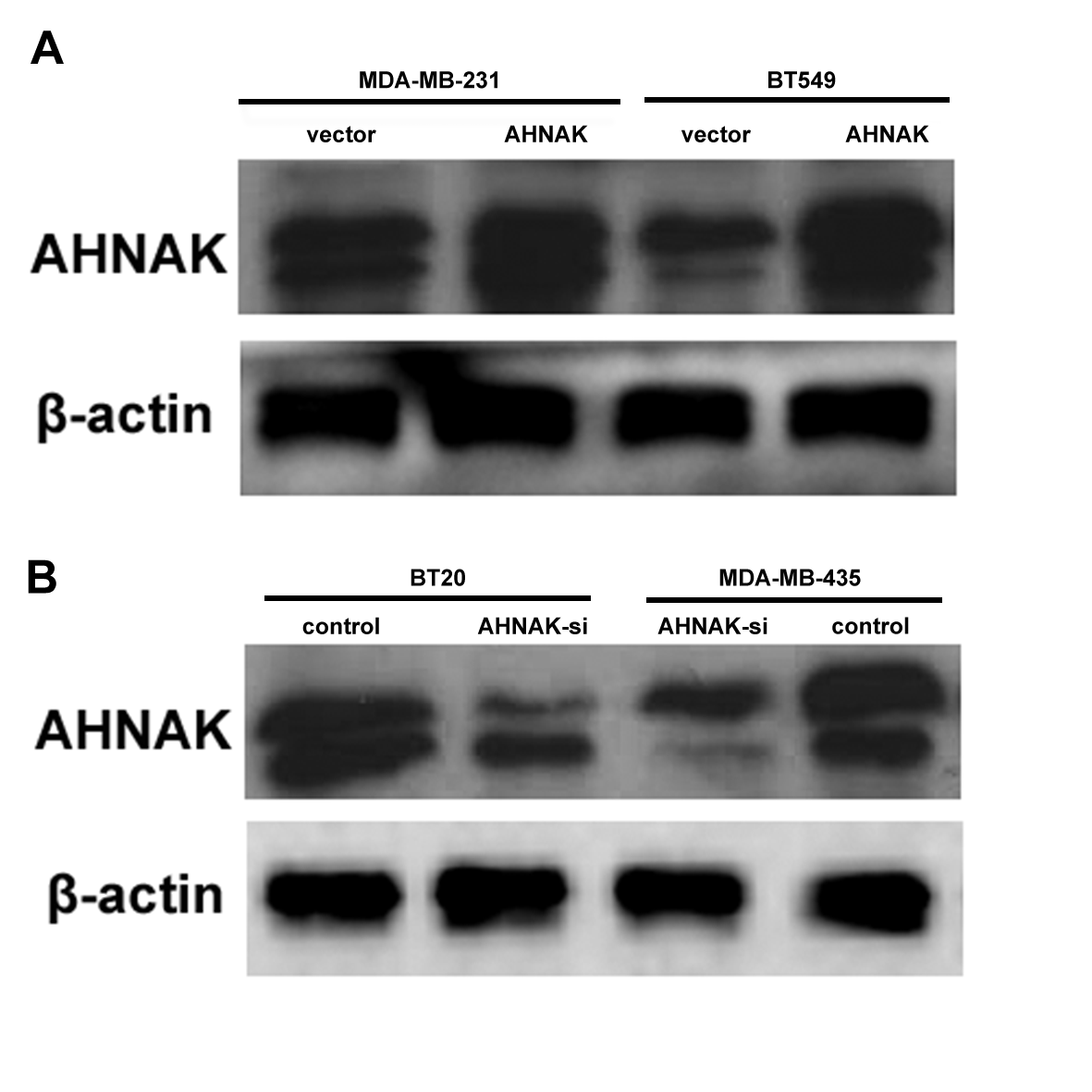

Supplement: Supplementary file 2 — Assessment of transfection efficiency by western blotting. (TIF 398 kb) [file 13046_2017_522_MOESM2_ESM.tif]
